# Physical Frailty and Fall Risk in Community-Dwelling Older Adults: A Cross-Sectional Study

**DOI:** 10.1155/2020/3964973

**Published:** 2020-07-04

**Authors:** Jiraporn Chittrakul, Penprapa Siviroj, Somporn Sungkarat, Ratana Sapbamrer

**Affiliations:** ^1^Department of Community Medicine, Faculty of Medicine, Chiang Mai University, Chiang Mai 50200, Thailand; ^2^Department of Physical Therapy, Faculty of Associated Medical Sciences, Chiang Mai University, Chiang Mai 50200, Thailand

## Abstract

**Introduction:**

Frailty is a condition in older adults with decreased physical and cognitive performance that can affect health outcomes associated with fracture, disability, and falls. The aim of this study was to compare fall risk with different physical frailty statuses and investigate factors associated with fall risk in community-dwelling older adults.

**Methods:**

The population studied included 367 older adults (mean age = 73.2 years ± 7.0; 237 females (64.6%) and 130 males (35.4%)) who live in Chiang Mai, Thailand. This study was of cross-sectional design. Fried's phenotype was used to screen the physical frailty status. The physiological profile assessment (PPA) was used to screen for fall risk. One-way ANOVA analysis was used to compare the fall risk between the different levels of frailty status. Linear regression analysis was used to assess the association between frailty status and fall risk.

**Results:**

The prevalence of the frailty group was 8.7% and that of the prefrailty group was 76.8%. The three statuses of frailty identified were found to have different levels of risk of falling. The frailty group had a higher fall risk than the nonfrailty group and the prefrailty group. In addition, the nonfrailty group had a lower fall risk than the prefrailty group.

**Conclusion:**

The frailty group had the highest fall risk in this cohort of older adults living in a community-dwelling facility. Therefore, it is important to assess the frailty status among older adults as it can be a predictor for fall risk. This assessment will therefore lead to a reduction in the rate of disability and death in the community.

## 1. Introduction

Frailty involves the concepts associated with the deterioration of the body related to the aging process. It encompasses the decline in physiology and biological syndromes of decreased reserve and resistance to stressors that lead to poor health outcomes such as loss of physical and mental performance [1–3]. Three clinical conditions are commonly used in the identification and classification of vulnerable older adults, specifically, comorbidity, frailty, and disability, which can lead to multiple adverse outcomes such as hospitalization and premature mortality in an aging population [4]. Nonetheless, there is no consensus regarding the prevalence rate of frailty across countries. In low-income and middle-income countries, there are indicators that the frailty prevalence in older adults is 12.7% in the frailty group and 55.2% in the prefrailty group [5]. Meanwhile, in Thailand, the frailty prevalence in the community-dwelling older adults was found to range from 15.0% to 17.2% [6, 7]. Currently, there are many methods used to assess frailty, of which Fried's frailty phenotype index is the most commonly used and currently has the highest levels of validity and reliability. It is mainly used as a unidimensional frailty index and can be used for both clinical and community assessment [8–10]. This index has five components consisting of unintentional weight loss, self-reported exhaustion, weakness (grip strength), slow walking speed, and low physical activity. Scores in three or more areas indicate the frailty group and in one to two areas indicate the prefrailty group, and if there are no scores, the classification is the nonfrailty group [8, 9].

In literature, a systematic review and meta-analysis showed frailty can predict future falls in community-dwelling older adults [11]. Previous studies indicated that a major outcome of frailty is falling [12–15]. Frailty and prefrailty are significant predictors of falls in older adults, and prefrail individuals have 1.36 higher odds of falling [16]. In addition, frailty causes decreased balance and mobility in older adults predicting falls within 12 months [17, 18]. According to the current knowledge (2016), frailty was associated with motor performance and the risk of falls in older adults [19]. Other previous studies reported that the older adults who had low muscle strength, weight loss, decreased gait speed, and high-level fear of falling were associated with frailty and falls [20, 21]. Meanwhile, fear of falling in older adults was related with low dual-task performance and reduced activity of daily living function [22]. In addition, there are differences in cognitive frailty between nonfrailty and prefrailty groups [23]. A prospective population-based study found that frailty and psychological and cognitive markers were associated with fall and fracture, increased recurrent falls and fractures, and decreased mobility [24, 25]. Likewise, frailty was associated with an increasing health perception level, a decline in the ability to adjust to serious incidents and respond to life events, a decrease in quality of life, and a decreased survival rate in older adults [26–28].

In older adults, falls are the second highest cause of injury-related deaths worldwide [29] and a significant factor which can lead to fracture, disability, and mortality [30]. People aged 65 and over comprise 28–35% of falls each year, a figure increasing to 32–42% in those over 70 years of age [29]. Falls are often associated with increase in age and frailty level [29]. The annual fall rate in older adults in Southeast Asia was found to be 6–31% in China and 20% in Japan [29] while in Thailand, in over-60-year population, it was 26.1%. Also, fall-related health problems accounted for 97.2% in the community [31].

Risk factors for falling are both intrinsic and extrinsic [32]. Intrinsic factors were gender, age, muscle weakness, gait and balance impairment, vision impairment, foot or ankle disorders, history of falling, fear of falling, polypharmacy, and medical conditions [33–35]. Extrinsic factors were home hazards, environmental hazards, inappropriate walking aids or assistive devices, footwear, and clothing [33, 35]. Intrinsic factors caused a higher frequency of falls than extrinsic factors, which led to greater levels of disability and mortality [36]. A previous study found that the fall rate associated with medical factors varied from 33.3% in cases of diabetes mellitus to 71.4% foot problems. Behavioural factors associated with a higher fall rate were underweight, abnormal balance, and gait [37].

Almost all studies assessed fall risk by mobility and physical performance tests to assess function and balance and timed up and go tests. Currently, the most frequently used tools of physical physiological fall assessment are the Balance Evaluation System Test (BESTest) [38] and the physiological profile assessment (PPA) [39] which indicate risk factors associated with falling. A systematic review reported that a multifactorial assessment of fall risk led to targeted intervention with efficient and effective strategies for preventing falls [40]. A previous study indicated that treatment of fall prevention in older adults should include an exercise program, home-safe interventions, vitamin D supplements, and multifactorial intervention [41]. Physiological profile assessment is a multifactorial assessment of fall risk which is used to evaluate a complete physiological assessment of fall risks in the older adults [39]. However, there is currently no study assessing fall risk among older adults with frailty using PPA.

Therefore, this study was designed to investigate any association between all physiological aspects and falls in frail older adults. The objective was to increase the level of information regarding the association between physical frailty status and difference in fall risk. Initial screening or assessment of fall risk in the older adults has been shown to prevent and reduce the risk of falling in a short space of time [42]. The aim of this study was to compare fall risk with different physical frailty statuses to understand more fully the different risk of falling in relation to each aspect of physiology at each level of frailty in older adults. This study also investigated factors associated with fall risk in community-dwelling older adults.

## 2. Materials and Methods

This study was cross-sectional in design. Community-based participants were recruited from the Saraphi District of Chiang Mai Province, Thailand. All participants gave written informed consent prior to inclusion. Ethical approval was given by the Human Research Ethics Committee of Medicine Faculty of Chiang Mai University (187/2018).

### 2.1. Participants

A power of population analysis was calculated from the total population of older adults in the Saraphi District using an alpha level of 0.05, the power was 95%, and the effect size was 0.5. The total population included 367 participants, 237 females (64.6%) and 130 males (35.4%), whose average age was 73.22 years ±7.00. The sample group selection was done by stratified random sampling from each group according to the population proportion of ten villages in the Khua Mung Subdistrict which meant a list of all older adults was sifted from a database made available by the community health center. The randomly selected population was chosen from a population of 804 people, aged 65 years or older, according to the following inclusion criteria: permanently residing in these villages and willing to participant in this study. Exclusion criteria were employed, following those advised in Fried's frailty phenotype [9] of disability and the physiological profile assessment [39]. These included severe audio and visual impairment or noncorrected audio and visual impairment, neurological disease (stroke and Parkinson's disease), and cognitive impairment using the Thai Mental State Examination (TMSE) enacted [43] by community medicine staff. The cut-point established for the TMSE defines cognitive impairment is ≤ 23 scores [43]. The individuals excluded from the sample were a single older adult with a current psychiatric diagnosis, sixty-eight older adults with disabilities, and one older adult with a current stroke diagnosis. Three eligible and randomized seniors refused to participate in the study. After exclusions, random sampling, and obtaining consent to the study, 367 older adults were recruited.

### 2.2. Data Collection

Questionnaire interviews were used to obtain demographic characteristics (age, sex, weight, height, and body mass index [44]), health history, medication, weight loss, and exhaustion questions. Weight and height were measured on the assessment day. We also collected physical frailty phenotype, and physiological profile assessment (PPA) estimated 40 min per participant.

### 2.3. Frailty Phenotype Assessment

This research used the five frailty phenotype criteria listed by Fried et al. [9] to assess frailty. The cut-off was 0 items (nonfrailty group), 1-2 scores (prefrailty group), and 3–5 scores (frailty group). These criteria included five components: (1) unintentional weight loss of >10 lb or ≥4.5 kg in the past year; (2) exhaustion evaluation using a two-question questionnaire which is derived from the Center for Epidemiological Studies Depression (CES-D) scale; interpretation was carried out using a total score equal to or greater than two points [45]; (3) low physical activity assessed by a modified international physical activity questionnaire which calculates kilocalories for one week (man > 383 and woman >270 kilocalories) [46]; (4) slow gait assessed by the overall walking time of the distance of 4.5 m; the interpretation was based on sex and height; (5) weakness measurement assessed with a grip strength dynamometer (Takei T. K. K. 5401 grip-D). Participants were measured in a standing position. Participants were asked to use their dominant hand and exert the greatest effort, performing the test three times. The highest possible value was elected and recorded in the results. Interpretation of the results utilized sex and body mass index.

### 2.4. Physiological Profile Assessment

The physiological profile assessment (PPA) has five component measures: visual contrast sensitivity, proprioception, quadriceps muscle strength, hand reaction time, and postural sway [39]. Visual contrast sensitivity was used to assess vision using the Melbourne Edge Test. The visual assessment is a test of the visibility of the intensity of a circular dividing line. The resulting score is the value of the last image seen [39]. The proprioception test was used to assess sensations using a lower limb matching test. The interpretation of the evaluation uses the difference in the degree of sensation in the big toes [39]. Quadriceps muscle strength was assessed using a spring gauge (kilograms) [39]. Hand reaction time was assessed using light as a stimulus and a finger depression of a switch as the response (milliseconds) [39]. Postural sway was assessed using the mass aggregation swing. The tested person stands on a foam sheet for 30 seconds, with a belt with a perpendicular nib, which draws a graph on the graph paper on the table while balancing. It takes the calculated graph from the anterior–posterior value, multiplies it by the medial-lateral value, and records the value [39]. *Z*-score was the standard of PPA fall risk score [39].

### 2.5. Statistical Analyses

The Shapiro–Wilk test was used to check normal distribution. Demographic data are presented as descriptive statistics. These included percentiles for ages, gender, number of comorbidities, polypharmacy, and frailty score. One-way ANOVA analysis was used to compare fall risk components with different frailty statuses. Multiple linear regression analysis was also used to investigate the factors associated with fall risk.

## 3. Results

The cohort included 367 community-dwelling older adults. The frailty group was 32 (8.7%), the prefrailty group was 282 (76.8%), and the nonfrailty group was 53 (14.4%). This study found the average of number of comorbidities was 0.83 ± 0.80 with a polypharmacy of 0.78 ± 0.81 and PPA fall risk (*Z*-score) of 2.80 ± 1.47. Body mass index (kg/m^2^) was 14.4% underweight, 39.0% normal weight, 36.5% overweight, and 10.1% obese (Table 1).

This study found differences in correlation between PPA fall risk and frailty status in all components. The visual contrast sensitivity components in the nonfrailty and the prefrailty groups were significantly higher than those in the frailty group. Proprioception components in the nonfrailty and the prefrailty groups were significantly lower than those in the frailty group. The knee extension strength components in the nonfrailty and the prefrailty groups were significantly higher than those in the frailty group. Hand reaction time components in the nonfrailty and the prefrailty groups were significantly lower than those in the frailty group. Posture sway components in the nonfrailty and the prefrailty groups were significantly lower than those in the frailty group. The PPA fall risks score (*z*-score) in the frailty group was significantly higher than those in the nonfrailty and the prefrailty groups (Table 2).

Multiple linear regression analysis found that frailty status (*B* = 0.71,95% CI = 0.42, 1.01), age (*B* = 0.07, 95%CI = 0.04, 0.09), and polypharmacy (*B* = 0.36, 95% CI = 0.00, 0.72) were associated with fall risk when adjusted by confounding factors such as age, sex, number of comorbidities, polypharmacy, and body mass index (Table 3).

**Figure 1:**
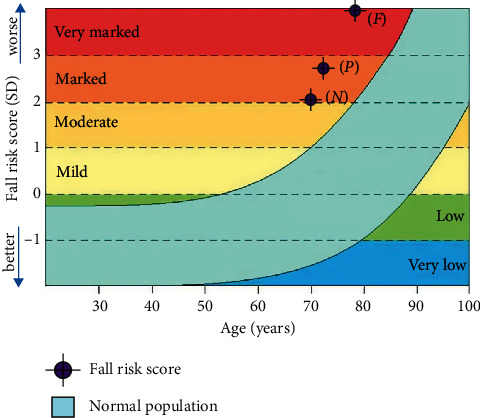
Overall fall risk score by frailty status and age using the fall risk calculator by NeuRA FallScreen®. *N* = nonfrail group (*Z*-score = 2.15); *P* = prefrail group (*Z*-score = 2.70); *F* = frail group (*Z*-score = 4.47).

Figure 1 shows an average of overall fall risk score by frailty status and age. The nonfrailty group has an age average of 70.60 years and an average overall score fall risk of 2.15. The prefrailty group has an average age of 73.20 years and an average overall score for risk of falls of 2.70. The frailty group has an age average of 79.31 years and an average overall score for fall risk of 4.47. The nonfrailty and prefrailty groups were at a marked level of fall risk, but the frailty group was at a highly marked level of fall risk.

## 4. Discussion

This study will add to the available evidence associated with the relationship between frailty in older adults and fall risk. The results of this study confirm our hypothesis that fall risk differs between each frailty status. Individuals classified as frail are at a greater risk of falling and then comes the prefrail and nonfrail groups. The study found that individuals classified as prefrail differ from those classified as nonfrail in three aspects of physiology, which is interesting new evidence.

The first set of results found that the frailty group had a significantly higher overall fall risk score when compared to both the nonfrailty and prefrailty groups. This is related to the level of frailty being related to the degeneration of physical and cognitive factors, conferring both physical frailty and cognitive frailty [23, 27].

Our study about fall risk had five components. First, these study results found the frail group had poorer vision than the nonfrailty group which was consistent with previous studies that found poor vision function in the frailty group caused falls in older adults [47–49]. This may be explained by the aging process leading to a change in focusing in the eyes leading to difficulty in focusing on distance or objects because of low contrast sensitivity [49, 50]. Frailty is also related to the concept of geriatric syndrome and age-associated reduction of physiological reserves [1–3, 51]. Second, the frailty group had impaired proprioception when compared with the nonfrail group. Likewise, a previous study found the frailty group had greater impaired proprioception than older adults not classified as frail [52]. Frailty is associated with a decline and change in physiology including the central nervous system (CNS) that serves to send sensations to the joints. This may explain this result as proprioception has cumulative neural input from mechanoreceptors such as muscular, articular, and cutaneous receptors [53]. Third, the frailty group had a more decreased muscle strength than the nonfrailty group which could be due to decreased muscle size and muscle mass from muscle fiber changes (IIA and IIB) producing lower strength in the frailty group [54]. Another study found a weakness of lower limbs was associated with the frailty group [55]. In addition, muscle weakness was found to be associated with the frailty group of older adults in community [56]. Fourth, this study found that the frailty group had a longer reaction time than the nonfrailty group which had not been reported in other studies. The explanation for this may be due to the reaction time being sensory in nature, responding to stimuli which reflected the speed impulses are passed to the central nervous system [57]. Reaction time in this study was assessed by hand reaction time that represents cognitive processes of the brain [58]. Similarly, frailty is associated with cognitive impairment which could also go some way to explaining the increase in the length of the reaction time [59]. Finally, this study found the frailty group has a greater postural sway than the nonfrailty group. Frailty is associated with reduced musculoskeletal and brain activity and both systems work together in coordinating posture stability. This study found the frailty group had a greater impairment of balance and gait [60] This therefore led to a high fall risk score (PPA *Z*-score) in the frailty group which was significantly different from the nonfrailty group.

In the second part of the study, we found the prefrail group to have proprioception differences compared with the nonfrail group. Basically, the prefrail group had a poorer proprioception than the nonfrail group. The results of this study are based on new knowledge, reflecting that the signaling changes in the brain were the first changes to occur before the frail condition became identifiable [53]. Likewise, the prefrail group had slower reaction times in comparison to the nonfrail group. This reaction time indicates a decline in cognitive function in the prefrail group showing a decline in brain function, again before entering the status of frailty. The members of the prefrail group also had lower muscle strength in comparison to the nonfrail group [59]. A previous study adds weight to this finding as it also found poor muscle strength and physical activity in a prefrail group when compared with nonfrail individuals [56]. In addition, a previous study found a reduction in mitochondrial genes in muscles which led to a reduction in muscle function [61]. The results of this study show that physiological changes before entering the frailty phase are a decrease in brain function and decrease in muscle strength which is interesting knowledge because it can be used as a way to prevent frailty in the future.

On the other hand, we found no differences in fall risk for two components between the nonfrailty and the prefrailty groups. These were vision and sway which may have occurred because the prefrailty group was nearly the same age as the nonfrailty group, and vision may be more closely related to age than frailty. Vision is the main component of postural control that affects postural sway [62, 63]. Thus, no difference in vision results in no postural sway differences for both statuses. In addition, we found the fall risk components proprioception and reaction time were no different between the frailty group and the prefrailty group. Those in the prefrailty group are likely to develop changes in physiological functions and progress to the frailty status. Sarcopenia is prevalent in the frailty group affecting neuromuscular changes that, together with those of greater age in the prefrailty group, will be related to loss of muscle mass and size which affect proprioception and reaction time [64].

In addition, we found frailty status, age, and polypharmacy were factors associated with fall risk. The meta-analysis found frailty was a risk factor for falling in community-dwelling older adults [15]. A prospective cohort study found a correlation between age and fall rate [32, 65]. A review of relevant literature showed polypharmacy to have a variable link to falling in older adults [66]. However another, nationwide nested case-control study found a direct relationship between polypharmacy and injurious falls [67].

This is probably the first study to separate the physiology of each aspect in assessing falls. However, our research showed some limitations that may have impact on the results. The first limitation of this study is that it was cross-sectional in nature. Second, the subgroup of frail individuals was too small. Finally, this study measured only the fall risk using physical performance assessment and there are many others factors associated with fall risk; therefore, in a future study, we would consider a prospective cohort design study.

## 5. Conclusions

This study found five different fall risk components associated with the frailty status. The frailty group had the highest fall risk score. In addition, the prefrail group was susceptible to changes related to physiology as regards proprioception, reaction time, and change in muscle strength, all of which were poorer than those in individuals in the nonfrail group. In addition, the frailty status, age, and polypharmacy were factors associated with fall risk which can be used to predict the risk of falling among older adults in the community. Thus, the older adults in the community should be screened for level of frailty and fall risk to reduce and prevent impact on disability and mortality. The results of our study can serve as a reference for specific intervention in the prevention of fall risk in community-dwelling older adults and also inform the assessment of other factors among community-dwelling older adults.

## Figures and Tables

**Table 1:** Characteristics of study participants.

Characteristics	Total	Frailty status (*N* = 367)			*p* value
		Nonfrail (*n* = 53, 14.4%)	Prefrail (*n* = 282, 76.8%)	Frail (*n* = 32, 8.7%)	
Sex, *n* (%)					0.641^a^
Male	130 (35.4)	20 (15.4)	101 (77.7)	9 (6.9)	
Female	237 (64.6)	33 (13.9)	181 (76.4)	23 (9.7)	
Age (years), mean ± SD	73.22 ± 7.00	70.60 ± 4.52	73.02 ± 6.95	79.31 ± 7.46	<0.001^b^
Number of comorbidities, mean ± SD	0.83 ± 0.80	0.75 ± 0.89	0.82 ± 0.79	0.97 ± 0.74	0.494^b^
Polypharmacy, mean ± SD	0.78 ± 0.81	0.64 ± 0.78	0.78 ± 0 .82	0.97 ± 0.74	<0.001^b^
Body mass index (kg/m^2^), mean ± SD	22.63 ± 3.86	22.50 ± 2.91	22.81 ± 3.90	21.24 ± 4.59	<0.001^b^
Underweight (<18.5), *n* (%)	53 (14.4)	4 (7.5)	38 (71.7)	11 (20.8)	
Normal weight (18.5–22.9), *n* (%)	143 (39.0)	25 (17.5)	109 (76.2)	9 (6.3)	
Overweight (23.0–27.5), *n* (%)	134 (36.5)	21 (15.7)	103 (76.9)	10 (7.4)	
Obese (˃27.5), *n* (%)	37 (10.1)	3 (8.1)	32 (86.5)	2 (5.4)	
PPA fall risk (*Z*-score) (mean ± SD)	2.80 ± 1.47	2.15 ± 1.02	2.70 ± 1.38	4.47 ± 1.65	<0.001^b^

^a^Chi-square test. ^b^One-way ANOVA test analysis. PPA = physiological profile assessment.

**Table 2:** Fall risk score using the physiological profile assessment compared with frailty status in older adults.

Component	Frailty status	Mean ± SD	95% confidence interval	*p* value
Visual contrast sensitivity (dB)	Nonfrailty^a^	17 ± 4.29	15.82, 18.18	<0.001^ac^^bc^
	Prefrailty^b^	16.10 ± 5.00	15.50, 16.68	
	Frailty^c^	8.44 ± 6.34	6.15, 10.72	

Proprioception (degree)	Nonfrailty^a^	2.29 ± 0.95	2.02, 2.55	0.001^ac ab^
	Prefrailty^b^	2.72 ± 1.49	2.54, 2.89	
	Frailty^c^	3.53 ± 1.99	2.81, 4.25	

Knee extension strength (kg)	Nonfrailty^a^	19.07 ± 8.07	16.84, 21.29	<0.001^ac ab bc^
	Prefrailty^b^	14.13 ± 6.32	13.39, 14.87	
	Frailty^c^	7.57 ± 4.40	5.98, 9.16	

Hand reaction time (ms)	Nonfrailty^a^	345.17 ± 94.99	318.99, 371.35	<0.001^ac ab^
	Prefrailty^b^	395.51 ± 136.49	379.51, 411.51	
	Frailty^c^	472.69 ± 228.53	390.29, 555.08	

Sway path (mm^2^)	Nonfrailty^a^	1216.24 ± 840.43	984.59, 1447.89	<0.001^ac bc^
	Prefrailty^b^	1367.63 ± 1258.65	1220.10, 1515.17	
	Frailty^c^	2435.25 ± 1951.94	1731.50, 3139.00	

PPA fall risk (*Z*-score)	Nonfrailty^a^	2.15 ± 1.02	1.87, 2.42)	<0.001^ac ab bc^
	Prefrailty^b^	2.70 ± 1.38	2.54, 2.86)	
	Frailty^c^	4.47 ± 1.65	3.87, 5.06)	

ac: nonfrailty group compared with the frailty group; ab: nonfrailty group compared with the prefrailty group; bc: prefrailty group compared with the frailty group; PPA = physiological profile assessment.

**Table 3:** Factors associated with fall risk.

Independent variable	Linear regression analysis		*p* value
	B (SE)	95% CI	
Frailty status	0.71 (0.14)	(0.42, 1.01)	<0.001^*∗∗*^
Age	0.07 (0.01)	(0.04, 0.09)	<0.001^*∗∗*^
Sex	0.26 (0.14)	(−0.01, 0.54)	0.06
Number of comorbidities	−0.28 (0.18)	(−0.65, 0.07)	0.12
Polypharmacy	0.36 (0.18)	(0.00, 0.72)	0.04^*∗*^
Body mass index	−0.01 (0.01)	(−0.05, 0.02)	0.42

^*∗∗*^Significance at *p*-value < 0.001.^*∗*^Significance at *p* value = 0.05.

## Data Availability

The data that support the findings of this study are available from the corresponding author upon reasonable request.
